# Hypereosinophilic endocarditis presenting with intracardiac mass and severe mitral regurgitation: a case report of FIP1L1–PDGFRA positive myeloid neoplasm

**DOI:** 10.1093/ehjcr/ytag296

**Published:** 2026-05-12

**Authors:** Nilay Sanjay Rao, Abhrajyoti Biswas, Shyam S Kothari, Pooja Vyas, Kewal Kanabar

**Affiliations:** Department of Cardiology, U. N. Mehta Institute of Cardiology and Research Centre (UNMICRC), Civil Hospital Campus, Asarwa, Ahmedabad, Gujarat 380016, India; Department of Cardiology, U. N. Mehta Institute of Cardiology and Research Centre (UNMICRC), Civil Hospital Campus, Asarwa, Ahmedabad, Gujarat 380016, India; Department of Cardiology, U. N. Mehta Institute of Cardiology and Research Centre (UNMICRC), Civil Hospital Campus, Asarwa, Ahmedabad, Gujarat 380016, India; Department of Cardiology, U. N. Mehta Institute of Cardiology and Research Centre (UNMICRC), Civil Hospital Campus, Asarwa, Ahmedabad, Gujarat 380016, India; Department of Cardiology, U. N. Mehta Institute of Cardiology and Research Centre (UNMICRC), Civil Hospital Campus, Asarwa, Ahmedabad, Gujarat 380016, India

**Keywords:** Case report, Loeffler’s endocarditis, Hypereosinophilic syndrome (HES), PDGFRA (platelet-derived growth factor receptor alpha), Imatinib, CEL (chronic eosinophilic leukaemia), TKI (tyrosine kinase inhibitors)

## Abstract

**Background:**

Loeffler’s endocarditis is a serious manifestation of hypereosinophilia, and it is associated with endomyocardial fibrosis, thrombus formation, valvular dysfunction, and, rarely, intracardiac mass lesions.

**Case summary:**

A 28-year-old male patient had progressive dyspnoea, facial oedema, and fatigue for 2 years. A 2D *trans*-thoracic echocardiogram revealed a 24 × 15 mm mobile echogenic mass attached to the mitral valve, associated with severe mitral regurgitation. Blood counts showed hypereosinophilia. Cardiac MRI showed subendocardial fibrosis. Bone marrow examination showed increased hypercellularity, and molecular testing was positive for FIP1L1–PDGFRA fusion, confirming a myeloid neoplasm with hypereosinophilia. The patient was treated with corticosteroids, anticoagulation, and Imatinib. Following initiation of therapy, the mass size reduced, and the patient improved symptomatically.

**Discussion:**

This case emphasizes the importance of a thorough diagnostic workup, including genetic testing in Loeffler’s Syndrome, to guide therapy. Recognizing molecularly driven eosinophilic disorders is essential, as targeted treatment can significantly improve prognosis. The presence of the Fip1-Like1-platelet-derived growth factor receptor alpha (FIP1L1–PDGFRα) fusion gene is a rare cause of hypereosinophilic syndrome requiring a distinct therapeutic approach.

Learning points

- Hypereosinophilic syndrome may uncommonly present with intracardiac unusual masses.
- Always test for PDGFRA rearrangements in patients with unexplained eosinophilia and cardiac involvement.
- Tyrosine kinase inhibitors induce rapid remission in PDGFRA-positive disease and improve prognosis.

## Introduction

Hypereosinophilic syndrome (HES) is defined as persistent eosinophilia (>1500/µL) and organ damage directly attributable to the HE.^1^ Cardiac manifestations such as mural thrombi, endomyocardial fibrosis, and restrictive cardiomyopathy can occur in up to 50% of HES patients.^1^ Secondary causes are more common and include parasitic or helminthic infections, allergic and atopic disorders, drug hypersensitivity reactions, autoimmune and vasculitic syndromes, connective tissue diseases, and paraneoplastic eosinophilia. Primary hypereosinophilia is associated with myeloid or lymphoid neoplasms that show eosinophilia and gene rearrangements involving PDGFRA, PDGFRB, FGFR1, JAK2, FLT3, or ABL1. It can also occur in myeloproliferative neoplasms such as CML and JAK2 V617F–positive MPN, acute myeloid leukaemia with inv (16) or t (16;16)/CBFB-MYH11, myelodysplastic syndromes with eosinophilia, MDS/MPN overlap syndromes, and aggressive systemic mastocytosis.^2^ A subset of cases is clonal and linked to genetic rearrangements, most notably FIP1L1–PDGFRA fusion, which characterizes a myeloid or lymphoid neoplasm with PDGFRA rearrangement.^2^ These patients respond strongly to tyrosine kinase inhibitors (TKIs), setting them apart from idiopathic or secondary HES.

We report a case of PDGFRA-positive eosinophilic disorder presenting with severe mitral regurgitation caused by Loeffler’s endocarditis, emphasizing the importance of molecular testing in suspected HES.

## Timeline of the case

**Table:** 

Timeline	Events
Day 1	A 28-year-old male with progressive dyspnoea, NYHA 3, oedema, fatigability, and weight loss for 2 years.
	CBC showed hypereosinophilia (72.3%), and other primary investigations were done.
	Echocardiography revealed a mobile echogenic mass attached to the mitral valve with severe mitral regurgitation.
Day 2	Investigated for secondary causes of HES.
	TEE shows severe MR with a mobile echogenic mass.
	HRCT of the thorax shows mosaic attenuation of the lung fields. No evidence of lung mass
Day 4	Secondary causes were negative. USG abdomen s/o splenomegaly. Work up for primary causes of HES.
Day 5	CMRI done s/o multifocal, patchy, Subendocardial LGE, patchy subepicardial LGE in mid to apical cavity inferior segments. LGE also noted at the anterolateral and posteromedial papillary muscles. Thickened both mitral valve leaflets with restricted posterior mitral leaflet mobility with severe MR.
Day 6	Bone marrow aspiration and biopsy s/o Hypercellular marrow with eosinophilia (36%), no dysplasia.
	Patient started on corticosteroid pulse therapy.
Day 9	Genetic study showed PDGFR ALPHA–positive for 1F1G signals consistent with FIP1L1–PDGFRA fusion.
	Patient started on Tab Imatinib 100 mg OD
Day 11	Patient discharged on tapering doses of oral corticosteroids and Tab Imatinib 100 mg OD.
On follow-up 6 months	Asymptomatic patient with an intra-cardiac mass resolved on echo with severe MR with reduction of eosinophilic counts to 9%. Patient is in a multidisciplinary team follow-up.

## Case Presentation

A 28-year-old male presented with a history of pedal oedema and exertional dyspnoea that has progressed from NYHA class II to III over the last 2 years. He also reported fatigue and palpitations that occur during exertion. The patient experienced significant weight loss over the past year. He works in the textile industry with chronic exposure to chemical fumes. There was no significant family history of cardiac disease. The patient was diagnosed with severe anaemia and eosinophilia 2 years earlier; however, no detailed investigations were conducted.

On examination, the vitals were a heart rate of 100 bpm, a blood pressure of 120/70 mmHg, and an oxygen saturation of 98% on room air. The jugular venous pressure was elevated and pulsatile. The patient had mild pedal oedema and splenomegaly. On cardiovascular examination, the patient had a holosystolic murmur suggestive of severe mitral regurgitation. Mild basilar crepitations were noted on respiratory examination.

**Figure 1:**
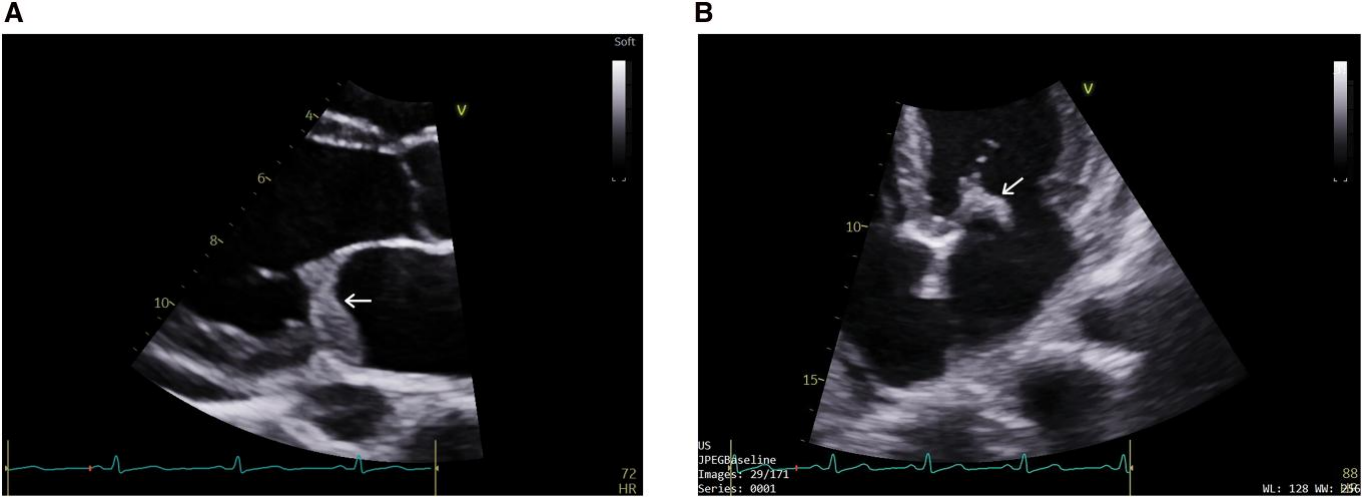
2D transthoracic echocardiography on presentation. (*A*) (Parasternal long axis view), (*B*) (A4C-Focused mitral valve) shows a large, mobile echogenic mass attached to the anterior leaflet of the mitral valve (marked by white arrow).

**Figure 2:**
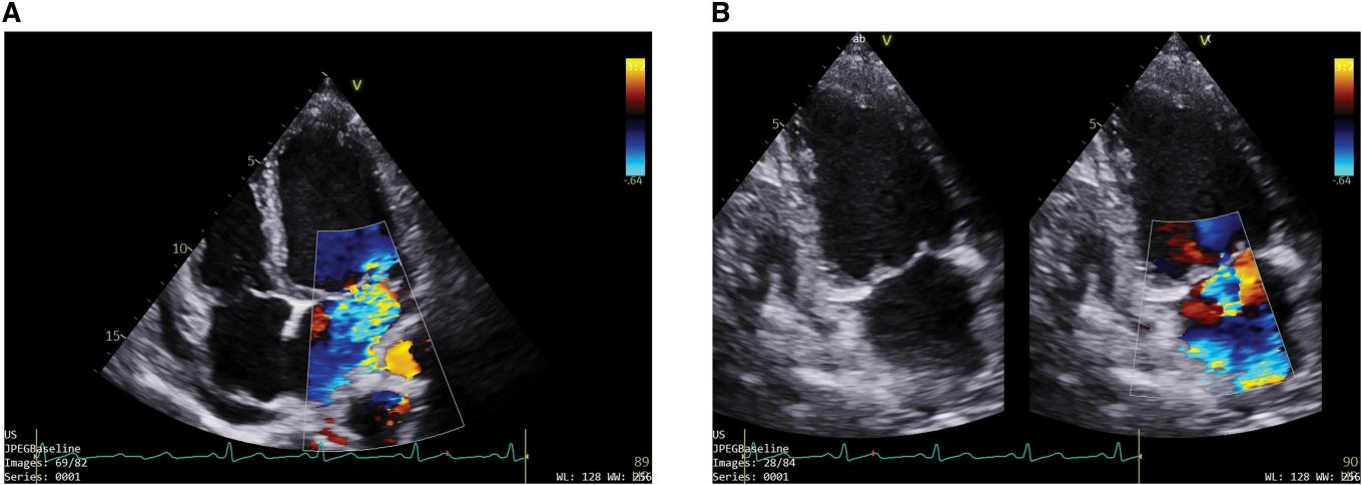
(*A*) Apical 4 chamber and (*B*) apical 2 chamber view shows severe mitral regurgitation with MRVC 7 mm with dilated LV size.

**Figure 3:**
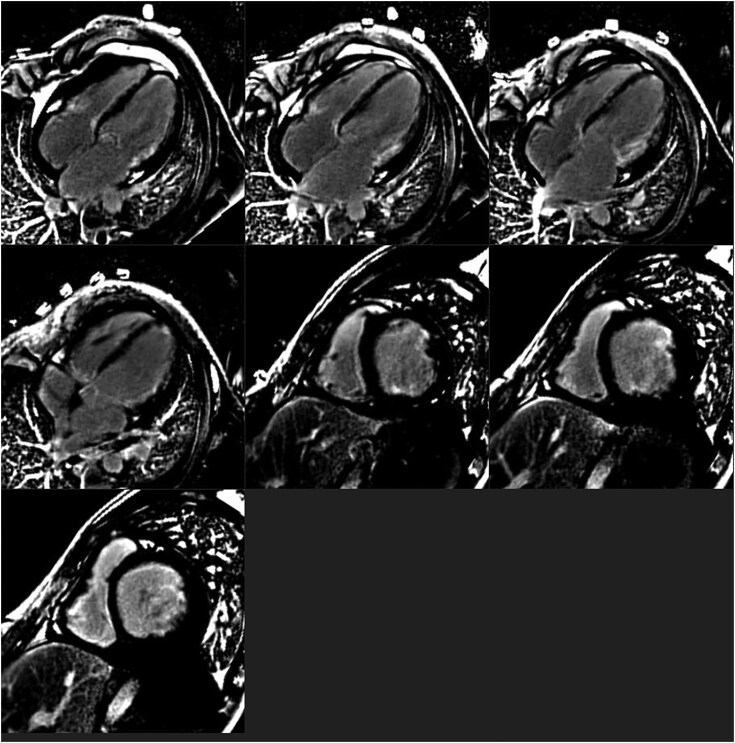
Cardiac MRI shows multifocal, patchy, subendocardial LGE, patchy subepicardial LGE in mid- to apical cavity inferior segments.

Complete blood count revealed a haemoglobin of 9 gm/dL with marked anisopoikilocytosis, normocytic normochromic red cells, and the presence of tear drop and target cells. The total leukocyte count was 21 770/cumm, with an absolute eosinophil count of 15 740/cumm (72.3%). Platelet count was 112 000/µL. The electrocardiogram showed a normal sinus rhythm; 2D transthoracic echocardiography demonstrated a large (24 × 15 mm), mobile echogenic mass attached to the mitral valve, severe mitral regurgitation (vena contracta 7 mm), and a dilated left ventricle with preserved systolic function, systolic pulmonary artery pressure- 69 mmHg, along with mild pericardial effusion (*Figures 1* and *2* and Supplementary Video 1–3). Cardiac MRI revealed a dilated left atrium with multifocal, patchy sub-endocardial and sub-epicardial late gadolinium enhancement (LGE) involving the mid- to the apical inferior segments, anterolateral and posteromedial papillary muscles, and thickened mitral valve leaflets with restricted posterior leaflet mobility, consistent with severe mitral regurgitation (*Figure 3*).

**Figure 4:**
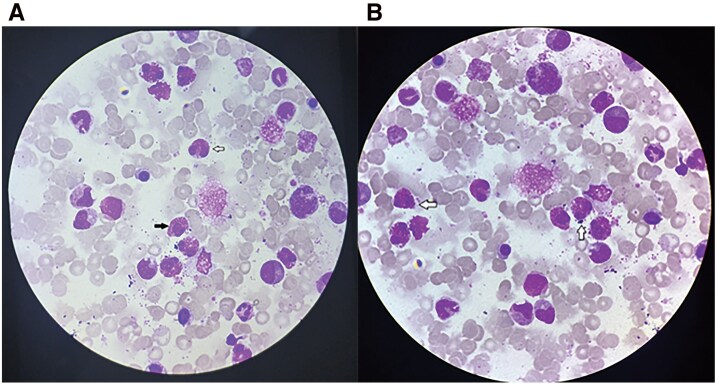
Bone marrow aspirate shows a marked increase in eosinophils (white arrow) and eosinophilic precursors (black arrow).

Bone marrow aspiration and biopsy showed a hypercellular marrow with 36% eosinophils, adequate megakaryocytes, and interstitial infiltrates of large-sized cells without dysplastic features (*Figure 4*). Molecular analysis demonstrated PDGFRA positivity in 53% of cells with 1F1G fusion signals consistent with *FIP1L1–PDGFRA* rearrangement, while *PDGFRB*, *BCR–ABL*, and *FGFR1* were negative. Blood cultures were negative. Serological evaluation negative for parasitic, autoimmune (ANA, C-ANCA, P-ANCA), and hypersensitivity pneumonitis panels. Serum IgE levels were 115 IU/mL (Normal value <100 IU/mL), while cardiac hs-troponin I levels were 44 pg/mL (Normal value <34.2 pg/mL in males). Stool examination was negative for ova, cysts, or parasites. HIV and HbsAg tests were negative, and renal and liver function tests were within normal limits.

The patient was initially treated with pulse methylprednisolone therapy for 3 days, followed by weight-based oral corticosteroids tapered weekly to suppress acute eosinophilic activity. Anticoagulation and guideline-directed medical therapy (GDMT), including beta blockers and ACE inhibitors, were started, and diuretics were added on top of it to maintain euvolemic status. After confirming PDGFRA gene positivity, targeted therapy with imatinib 100 mg daily was initiated.

**Figure 5:**
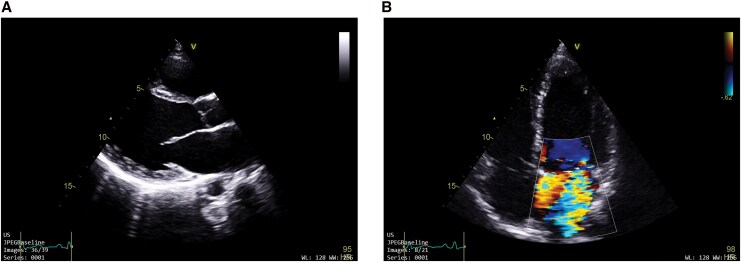
(*A*) (parasternal long axis view) follow-up echocardiography showed resolution of mass. (*B*) (Apical 4 chamber view) Follow-up echocardiography showed severe mitral regurgitation.

Following the initiation of corticosteroids and imatinib, the patient’s symptoms improved from NYHA class III to NYHA class I over 6 months, and follow-up imaging showed a reduction in the size of the mass attached to the mitral valve, with persistent severe mitral regurgitation (*Figure 5*). Systolic pulmonary artery pressure was 34 mmHg. During the 6-month follow-up, the patient was asymptomatic and achieved a complete haematologic response with an eosinophil count of 9%. The patient was advised to undergo bioprosthetic mitral valve replacement, but declined surgery due to his asymptomatic status. The long-term management strategy for this patient is to continue clinical follow-up and ensure adequate suppression of eosinophilia.

## Discussion

This case highlights the diagnostic and therapeutic importance of identifying PDGFRA-positive eosinophilic neoplasms. The FIP1L1–PDGFRA gene rearrangement is an uncommon yet well-recognized cause of hypereosinophilia. It occurs due to a 4q12 chromosomal deletion that fuses the FIP1L1 and PDGFRA genes, resulting in a constitutively active tyrosine kinase that promotes the proliferation of eosinophilic cells. This fusion gene has been observed in approximately 10% to 60% of hypereosinophilia cases and shows a strong male predominance (around 95%).^3,4^

The clinical presentation of HES is highly variable and largely depends on the organs affected. Reported organ involvement varies widely, from 19% to 91% of cases. The most commonly affected systems include the skin (32%–57%), spleen (44%–52%), and lungs (30%–45%).^3,4^ General symptoms such as fatigue and weight loss are also common, occurring in approximately 62% and 12% of patients, respectively.^4^ Cardiac involvement varies, with an incidence ranging from 19% to 34%, and is associated with significant mortality due to extensive lesions and progression to heart failure.^2–4^ Cardiac manifestations include endocardial fibrosis, mural thrombi, and restrictive physiology. Cardiac involvement, especially endomyocardial fibrosis, is a key prognostic factor in HES, with recent data indicating a prevalence of 22% in F/P+ CEL.^3^ Other types of cardiovascular involvement include constrictive pericarditis, endomyocarditis, valvular dysfunction, and myocardial infarction.^5^

Our patient presented with a predominant valvular mass and severe MR, which is less commonly described. Valvular insufficiency in HES is commonly related to mural endocardial thrombosis, fibrosis involving the leaflets or chordal thickening of the mitral or tricuspid valves.^5^ Persistence of severe MR is due to fibrotic remodelling of the mitral valve. A rare case of HES with acute mitral regurgitation due to papillary muscle rupture has been reported in the literature.^6^

Imatinib is highly effective, achieving complete haematologic and molecular remission in most patients.^7^ Steroids may be used initially for rapid eosinophil suppression, but long-term management depends on TKIs. After TKI therapy, the 5-year survival rate of 93.5% was reported in a single Chinese centre.^8^ Severe valvular regurgitation may require surgery despite haematologic remission. Potential indications of surgical correction are symptomatic severe MR (NYHA III, IV) with symptoms attributable to MR, progressive LV dysfunction, recurrent pulmonary oedema and heart failure hospitalizations, severe MR with pulmonary hypertension, and recurrent thromboembolism. Although we found a single case report describing the reversibility of severe mitral regurgitation after pharmacological treatment of HES, mitral valve replacement was not needed.^9^ HES patients have a high tendency for prosthetic heart valve thrombosis; hence, bioprosthetic valves are preferred to mechanical valves.^10^

This case demonstrates PDGFRA-positive hypereosinophilic syndrome presenting as Loeffler’s endocarditis with severe mitral regurgitation. It underscores the importance of molecular testing in unexplained eosinophilia with cardiac involvement. Early initiation of imatinib can dramatically improve outcomes, but surgery may be necessary for advanced valvular disease.

## Supplementary Material

ytag296_Supplementary_Data

## Data Availability

The data underlying this article will be shared on reasonable request to the corresponding author.

## Lead author biography

MBBS, MD MedicineResident in DM CardiologyU N Mehta Institute of Cardiology and Research Centre, Ahmedabad, India.

## Notes

**Consent:** The authors confirm that written consent for submission and publication of this case report including images and associated text has been obtained from the patient in line with COPE guidance.
